# Molecular Detection and Genetic Characterization of *Ehrlichia ruminantium* Harbored by *Amblyomma hebraeum* Ticks of Domestic Ruminants in North West Province, South Africa

**DOI:** 10.3390/ani12192511

**Published:** 2022-09-21

**Authors:** Sifiso S. Mnisi, Malekoba B. N. Mphuthi, Tsepo Ramatla, Lehlohonolo S. Mofokeng, Oriel Thekisoe, Michelo Syakalima

**Affiliations:** 1 Department of Animal Health, School of Agricultural Sciences, North-West University, Private Bag X2046, Mmabatho 2735, South Africa; 2 Unit for Environmental Sciences and Management, North-West University, Private Bag X6001, Potchefstroom 2531, South Africa; 3 Disease Control Department, School of Veterinary Medicine, University of Zambia, Lusaka P.O. Box 32379, Zambia

**Keywords:** heartwater, *Amblyomma hebraeum*, tick, *Ehrlichia ruminantium*, genotype, domestic ruminants

## Abstract

**Simple Summary:**

Bont ticks (Genus *Amblyomma*) transmit *Ehrlichia ruminantium* (*E. ruminantium*), a pathogen that causes a rickettsial disease called heartwater in ruminants. Heartwater disease is devastating and causes financial loss in livestock farming in most parts of southern Africa. The *E. ruminantium* tends to have many variants; as a result, it is difficult to develop vaccines for controlling it. Knowledge of *E. ruminantium* variant occurrence in a particular area may assist in designing appropriate vaccination control strategies. Thus, this study investigated the *E. ruminantium* variants found in the Ngaka Modiri Molema District, North West Province, South Africa. Diversity of these *E. ruminantium* variants was determined by PCR and sequencing of DNA pools extracted from the tick vectors. Six different DNA variants of *E. ruminantium* were detected and compared well with those known to be virulent from other parts of South Africa and other countries. The type of variant commonly used for the massive vaccination was not detected in the study area.

**Abstract:**

*Ehrlichia ruminantium* (*E. ruminantium*) is the causative agent of heartwater disease and it is mainly transmitted to livestock by *Amblyomma hebraeum* (*A. hebraeum*) tick in South Africa. This study investigated the occurrence of *E. ruminantium* and its genetic diversity in ticks within Ngaka Modiri Molema district of North West Province in South Africa. Genomic DNA was extracted from whole *A. hebraeum* ticks totaling 876 and resulted in a total of 292 pooled DNA samples. Firstly, conventional PCR was used to detect *Ehrlichia* spp. targeting the *dsbA* gene, followed by nested PCR targeting the *Map1* gene performed on DNA pool samples that were positive from the first PCR. One hundred and six tick DNA pool samples were positive by *dsbA* gene PCR for the presence of *Ehrlichia* spp. with minimum infection rate (MIR) of 121, while 13/106 were positive by *Map1* PCR with MIR of 15. Different *E. ruminantium Map1* genotypes (NWUe1, NWUe2, NWUe3, NWUe5, and NWUe6) were detected from tick samples and were closely related to more than 13 gene sequences of *E. ruminantium* from the NCBI GenBank database. These findings suggest that there is a significant diversity of *E. ruminantium* infecting ticks in the study area.

## 1. Introduction

*Amblyomma hebraeum* (Acari: Ixodidae) is a vector of *Ehrlichia ruminantium* that causes heartwater, which is among problematic diseases affecting domestic ruminants in southern Africa [1]. In South Africa, the *A. hebraeum* tick is found along the coastal belt of the Eastern Cape Province, through KwaZulu-Natal, Gauteng, Mpumalanga, North West, and Limpopo provinces [2]. It has also been identified in southern African neighboring countries, such as Botswana, Zimbabwe, Mozambique and Eswatini [2]. *Ehrlichia ruminantium* is a member of the order Rickettsiales and is the etiological agent of heartwater disease in domestic ruminants (cattle, sheep, goats) as well as a wide range of wild ruminants [3]. The *E. ruminantium* is characterized by a number of virulent genotype strains, namely, Welgevonden, Gardel, Ball3, and Mara 87/7, to name a few. Extensive gene recombination seems to take place among genotypes of *E. ruminantium,* which suggests that recently recombined strains are continuously developing in the field. This is likely to happen when organisms are extracellularly within the tick immediately after bloodmeal before intracellular infection is established [4,5]. A recent study that was done by Steyn and Pretorius [1] in South Africa from seven provinces that are endemic to heartwater disease has identified 25 new genotypes, and they also stated that recombination is also possible when either the mammalian or the vertebrate host is infected with more than one isolate or when two infected ticks with two different isolates feed on the same animal. The *E. ruminantium* Welgevonden strain is the most virulent in South Africa [6] and the Ball3 strain is found in the infective blood vaccine. 

However, there is scarcity of data on how widely distributed is this *E. ruminantium* Welgevonden strain and others in other parts of South Africa. On the other hand, the variation and virulence of the genotype strain has an effect on the vaccine efficacy. This lack of information, therefore, increases the necessity for studies to investigate the distribution of such strains in order to devise policy and control measures for the disease. The study that was previously done by Steyn and Pretorius [1] did not include the Ngaka Modiri Molema District in the North West Province.

The aim of this study was to detect occurrence and to conduct genetic characterization of *E. ruminantium* strains circulating in ticks collected from domestic ruminants within the Ngaka Modiri Molema District of the North West Province, South Africa.

## 2. Materials and Methods

### 2.1. Study Area

**Figure 1:**
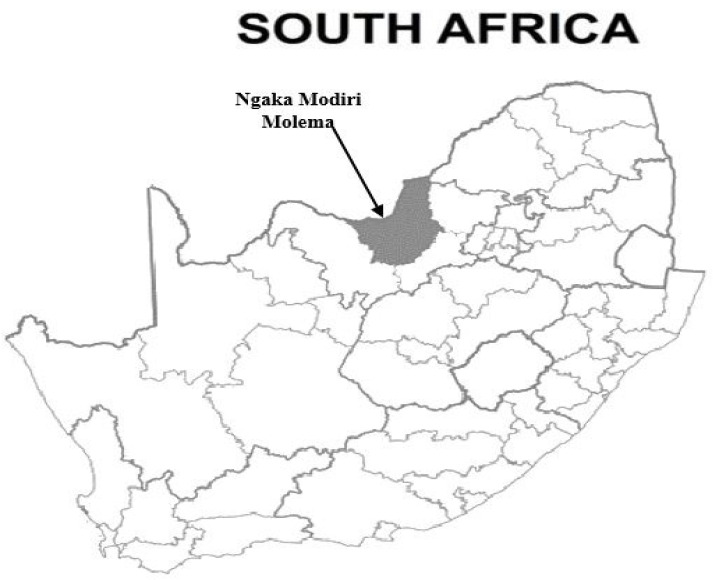
Map indicating the sampling location (Map made with ArcGIS) in the North West province in Ngaka Modiri Molema District Municipality.

Ngaka Modiri Molema District Municipality (Figure 1) covers a surface area of 28,206 km^2^. It is one of the four district municipalities in the North West Province. It is situated centrally within the province and shares an international border with Botswana. The district comprises five local municipalities, namely, Mahikeng, Ratlou, Ramotshere Moiloa, Ditsobotla, and Tswaing. Samples were collected across the whole district, with the different municipalities as the focal centers of sampling. Sampled animals were from communal grazing systems and were intentionally targeted since livestock in those areas are rarely dipped for tick control and are also rarely vaccinated. 

### 2.2. Tick Collection 

*Amblyomma hebraeum* ticks were collected from livestock throughout all seasons between the years 2017 and 2018. Ticks were collected from cattle in the hairless parts of the animals, at the base of the tail and around the anus, axilla, sternum, belly, and groin. In small stock (sheep and goats), in addition to the above sites, they were also collected in interdigital spaces of hooves.

Ticks were removed from the host using forceps, and then placed in a suitable container and preserved with 70% alcohol until used. The specimens were morphologically identified using a tick identification literature of Walker [7] on a stereo microscope (Nikon, c-leds). An expert entomologist from the Agricultural Research Council–Onderstepoort Veterinary Research (ARC-OVR) also confirmed the identity of the ticks. Genomic DNA pool samples were further amplified by PCR, sequenced, and compared to sequences in GenBank using BLASTn analysis to supplement morphological species identification. 

### 2.3. Processing of Samples 

A total of 876 ticks were washed in sterile water and processed as pooled samples with an average of 3 ticks per sample based on the site area of collection, same host, and sex (male or female). The engorged adult ticks were included for DNA extraction in this study as well. As a result, DNA was extracted from 292 pooled samples using the DNeasy^®^ Blood & Tissue kit (Qiagen, Germany) according to the manufacturer’s instructions. The quality and quantity of the extracted DNA was analyzed using spectrophotometry with a NanoDrop ND-100 system (NanoDrop Technologies, Inc., Wilmington, NC, USA). Spectrophotometrically, DNA purity ratio at 260 and 280 nm (A_260_/A_280_) absorbance was determined. Pure quality DNA is between 1.7 and 2 ratios. Thereafter DNA samples were stored at −70 °C until used for PCR analysis.

### 2.4. Molecular Identification of Ticks

PCR targeting of the internal transcribed spacer 2 (ITS2) gene fragment was conducted for further identification of the ticks. This was done on all the tick DNA pools and 8 representatives of positive amplicons were sequenced. Primers used were, ITS2F forward (YTG CGA RAC TTG GTG TGA AT) and ITS2R reverse (TAT GCT TAA RTT YAG SGG GT) and were employed as previously described by Muruthi [8]. The PCR reaction mixture had a final volume of 25 µL, which consisted of 12.5 μL of AmpliTaq Gold 360^®^ Master Mix (Applied Biosystems, Foster City, CA, USA), 1 μL each of primer [each at 10 μM concentration], 2 μL of the template DNA, and 8.5 μL double distilled water. PCR conditions consisted of initial denaturation at 95 °C for 10 min, 35 cycles of denaturation at 95 °C for 30 s, annealing at 47 °C and 50 °C for 30 s, and extension at 72 °C for 60 s, followed by a final extension at 72 °C for 7 min and final hold at 4 °C, using the Bio-Rad T100™ Thermal cycler (Bio-Rad, Foster City, CA, USA). Nuclease-free water was used as a non-DNA negative control and positive control (Haemaphysalis longicornis DNA) obtained from Obihiro University, Japan. 

### 2.5. Detection of Ehrlichia Species from Amblyomma Ticks Using Conventional PCR

PCR was performed in a Bio-Rad T100™ Thermal cycler (Bio-Rad, Hercules, CA, USA) according to the guidelines described by Iweriebor et al. [9] for amplification of the genus-specific disulfide bond formation protein (*dsbA*) gene using the following universal primers: EHL *dsb* F 5′-GAT GAT GTC TGA AGA TAT GAA ACA AAT-3′ and EHL *dsb* R 5′-CTG CTC GTC TAT TTT ACT TCT TAA AGT-3′ to generate 409 bp fragments. PCR was performed in a 25 μL reaction mixture containing 12.5 μL of One Taq^®^ quick-load^®^ master mix (New England BioLabs, Ipswich, MA, USA), 1 μL of 10 pMol for each of the forward and reverse primers, 8.5 μL of nuclease-free water, and 2 μL of DNA template. The cycling conditions were as follows: an initial heating block at 94 °C for 3 min, followed by 35 cycles of denaturation at 93 °C for 30 s, then annealing at 47 °C for 30 s, with an elongation at 72 °C for 1 min and a final elongation at 72 °C for 5 min. Nuclease-free water was used as a non-DNA negative control and positive control was *E. ruminantium* DNA obtained from a collaborator at the Center for Zoonosis Control, Hokkaido University, Japan. PCR products were visualized by electrophoresis on a 1% agarose gel stained with ethidium bromide at 100 volts for 45 min. Positive PCR products were sent to Inqaba Biotech (Pty), Ltd., Pretoria (South Africa) for purification and sequencing. 

### 2.6. Determination of Genetic Diversity of Ehrlichia Species by Analysis of the Map1 Gene

Nested PCR targeting of the *Map1* gene was performed on DNA pool samples that were positive from the first PCR (detection of *Ehrlichia* spp.) to determine the diversity according to Esemu at al. [10]. The primers used to detect the *Map1* gene are shown in Table 1.

The individual reaction mixture for the primary round of PCR consisted of template DNA (2 μL), 12.5 μL DreamTaq PCR master mix (2X) (Thermo Scientific), and 1 μL of individual primer from a working solution of 20 μM (final concentration of 0.4 μM) and 8.5 µL of nuclease-free PCR water to yield a 25-μL total individual reaction volume.

DNA pool amplification were carried out in a Bio-Rad T100™ PCR thermal cycler (Bio-Rad, USA) under the following cycling conditions: for the first round of PCR reaction, initial denaturation was done at 94 °C for 3 min, followed by 35 cycles of denaturation (94 °C for 45 s), primer annealing (53 °C for 45 s), and primer extension (72 °C for 45 s). The final extension was done at 72 °C for 10 min and the reaction stopped by cooling to 4 °C until the samples were collected. For the second round of PCR amplification, aliquots of 1 μL of PCR product from the first round of PCR amplification were used as the DNA template. The PCR amplification conditions for the second round were as follows: initial denaturation at 94 °C for 3 min, followed by 40 cycles of denaturation (94 °C for 1 min), primer annealing (57 °C for 1 min) and primer extension (72 °C for 1 min). The final extension was at 72 °C for 10 min and holding at 4 °C until the samples were collected. The expected PCR product size after the second round of PCR amplification was an 800 bp partial fragment. Each batch of PCR run included one negative control of sterile distilled water and one positive control as a template. Positive PCR products were sent to Inqaba Biotech (Pty), Ltd., Pretoria (South Africa) for purification and sequencing.

### 2.7. Sequence Analysis of Map1 PCR Products and Phylogenetic Tree Construction

High-quality sequences were then subjected to analysis and alignment with MAFFT program 6.864 for similarity matches between the *E. ruminantium* strains in the present study and reference strains retrieved from GenBank [11,12]. Similarity matches between *E. ruminantium* strains in the present study were determined in a pair-wise sequence alignment using BioEdit version 7.0.9 [13]. 

A phylogenetic relationship was conducted with MEGA6, whereby the neighbor-joining (NJ) and distance matrix methods were used [14]. The numbers at the nodes correspond to bootstrap values accessed with 1000 replicates. Evolutionary analyses were conducted in MEGA6 [15]. Manipulation and tree editing was carried out using Tree View [16]. An *Ehrlichia canis* sequence was used as an outgroup.

### 2.8. Ethical Consideration

This study was approved based on animal research ethics committee guidelines by the North West University Research Ethics Regulatory Committee (NWU-RERC) Ethics Number: NWU-00358-19-A5. Consent was also obtained from the animal owners before collection of samples.

## 3. Results

### 3.1. Tick Samples and Their Identification

Convenience sampling was used, and a total of 876 adult *Amblyomma hebraeum* ticks were collected from communal villages of Ngaka Modiri Molema District. However, in the Tswaing Municipality, there were no *A. hebraeum* ticks during the period of sampling (Table 2). 

The BLASTn results for the ITS2 gene fragment of *Amblyomma* spp. sequences from this study matched with *A. hebraeum* sequences with a percentage identity of 99%. The *A. hebraeum* representative nucleotide sequences were deposited on the GenBank database under accession numbers ON888429, ON888430, ON888431, ON888432, ON888433, ON888434, ON888435, and ON888436. The phylogenetic tree was constructed to illustrate and further confirm that the *A. hebraeum* of the current study are genetically similar (share same clade) to relevant species from other areas/countries in comparison from other species in the same genus (Supplementary Figure S2).

#### 3.1.1. Amplification of Ehrlichia spp. Using PCR 

A total of 106 out of 292 tick DNA pool samples were positive by *dsbA* gene (409 bp fragments) PCR. Fifteen representative (Bovine = 7, Ovine = 6, and Caprine = 2) sequenced amplicons had an identity of 98–100% with *Ehrlichia ruminatium* (Table S1). Table 2 shows the sampling areas within the Ngaka Modiri Molema District with their total number of ticks collected and total number of positive samples for *Ehrlichia* spp. The representative nucleotide sequences for *E. ruminantium* were deposited on the GenBank database under accession numbers ON994658, ON994659, ON994660, ON994661, ON994662, ON994663, ON994664, ON994665, and ON994666.

#### 3.1.2. Determination of Genetic Diversity of E. Ruminantium Strains from A. Hebraeum Ticks through Analysis of the *Map1* Gene 

Thirteen samples were positive for *Ehrlichia* spp. By *Map1* PCR. Five *Map1* gene amplicons produced good quality sequences. All 5 positive *Ehrlichia* spp. *Map1* gene sequences were given identity codes (NWUe1, NWUe2, NWUe3, NWUe5, and NWUe6) and were then subjected to BLASTn. The *Map1* gene sequences from this study shared identity with some *E. ruminantium* strains from GenBank as shown in Table 3. The identity among the *Map1* nucleotide sequences from the current study ranged from 98.7 to 100%. 

The NWUe1 shared identity with South African (99.7%) and Zimbabwean (99.5%) strains from the GenBank with the accession numbers AF125274.1 and AF125277.1 respectively with query coverage of 99.0%. The NWUe2, on the other hand, shared identity with strains from South Africa (99.0%), Zimbabwe (98.6%), and Burkina Faso (98%), with accession numbers AF368000.1, U50834.1, and AF368001.1, respectively, with query coverage of 98.0–99.0%. The NWUe3 was 99.6% identical to *E. ruminantium* strain from Gambia (accession number EF627980.1), France (CR925678.1), and three from South Africa (AY343331.1, CR767821.1, and U49843.1), with query coverage of 99%. NWUe5 shared identity with *E. ruminantium* strains from Southern Africa (AF368000.1 [100%]), Nyatsanga strain [Eastern Caribbean islands and Africa] (U50834.1 [99.6]) and Western Africa (AF368001.1 [98.8%]), while the query coverages were 97%, 99%, and 99%, respectively. Lastly, the NWUe6 also shared a 98% identity with *E. ruminantium* strains from Zimbabwe (AF125274.1) and (AF125277.1), with both having query coverage of 99% (Table 3). 

### 3.2. Phylogenetic Tree Construction of Ehrlichia spp. Based on Map1 Gene

**Figure 2:**
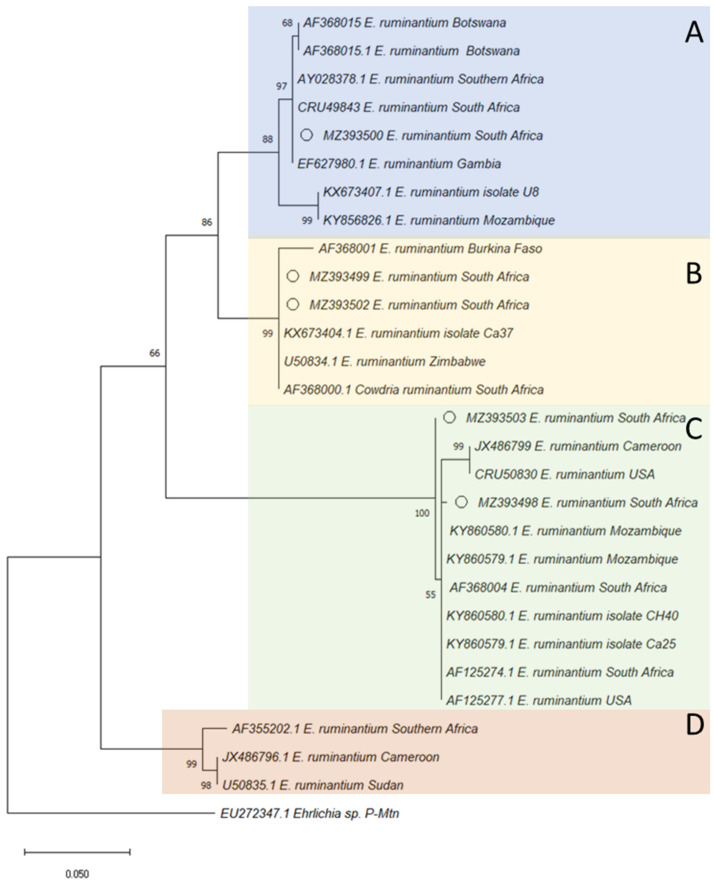
Phylogenetic tree based on the sequences of the *E. ruminantium MAP1* gene. The tree was constructed using MEGA based on the maximum likelihood method, using the T92+G Model. The number at nodes correspond to bootstrap values accessed with 1000 replicates, and the analysis consisted of 504 bp aligned sequences. Five nucleotide sequences were used for data analysis. Sequences of this study are indicated by a circle bullet. The sequences that generated (**A**) Blue, (**B**) Yellow, (**C**) Purple and (**D**) Orange are 3 different clusters. The sequence from this study are indicated by open circles.

The *Ehrlichia* spp. *Map1* sequences obtained in the current study were positioned in three different clusters which are referred to as A, B, and C (Figure 2). Two *Map1* nucleotide sequences from this study (MZ393502 and MZ393499) were positioned in cluster A and grouped with 2 *Map1* sequences from Zimbabwe (U50834.1 and KX673404.1), and another from South Africa (AF368000.1). In cluster B, the *Map1* sequence (MZ393500) was grouped with sequences from Gambia (EF627980.1), and South Africa (CR49843.1 and AY028378.1). On the other hand, *Map1* sequences (MZ393498 and MZ393503) in cluster C were grouped with 2 *Map1* nucleotide sequences from Zimbabwe (KY860579.1 and KY860580.1) and 1 from South Africa (AF368004.1) (Figure 2). 

## 4. Discussion

The *Ehrlichia* spp. DNA was detected from ticks collected from livestock in 4 out of 5 municipalities of the Ngaka Modiri Molema District. PCR for amplification of *DsbA* generated MIR of 121 of *Ehrlichia* spp. from *A. hebraeum* ticks. Mahikeng Municipality had 141 MIR of infected ticks, followed by the Ramotshere Moiloa, Ratlou, and Ditsobotla municipalities with 130, 97, and 58 MIR respectively. There were no *A. haebreaum* tick specimens found during tick collection in the Tswaing Municipality. Our results are not unusual because a tick survey done earlier by Spickett et al. [17] also showed a very low prevalence of *Amblyomma* ticks in Tswaing Municipality. That study further reiterated that *Amblyomma* ticks are commonly found in the north-eastern and northern Bushveld regions of the North West Province. 

The *Ehrlichia* spp. positively detected by PCR were 121 and 15 per MIR for *dsbA* gene and *Map1* gene, respectively. These findings show that the *dsbA* gene PCR was more sensitive for detection of *Ehrlichia* spp. DNA when compared to the *Map1* gene PCR for tick DNA samples. However, it has been suggested that the *Map1* gene may be an ideal target for classification and genotypic characterization of these *Ehrlichia* spp. as supported by Matos et al. [18]. It should be noted that detection of *Ehrlichia* spp. DNA from ticks does not necessarily mean infection on all as some could still be harboring infected blood from the host. 

The *E. ruminantium* has a *Map1* multigene family that consists of 16 copies of homologous genes encoding 28–30 kDa outer membrane proteins and are highly diversified among strains of *E. ruminantium* [19]. This study was able to find a highly prominent degree of genetic sequence variation, hence, the *E. ruminantium* of the current study falls within 3 different clusters on the phylogenetic tree which is in conformity with previous reports [18,19,20]. In this study, the *Map1* nucleotide sequences of *E. ruminantium* NWUe2 and NWUe5 were detected from cattle ticks in the Mafikeng Municipality, NWUe1 and NWUe6 from cattle ticks in Ramotshere Moiloa Municipality, and NWUe3 from cattle ticks in Ratlou Municipality. Above results showed that the *E.* *ruminantium* variants of this study are genetically diverse based on sampled municipal area, hence they are scattered in three different clusters (A, B, and C) of the phylogenetic tree. The NWUe1 and NWUe6 nucleotide sequences were identical to *E. ruminantium* Welgevonden (AF125272.1) and LemcoT3 (AF125277.1) strains. 

The Welgevonden genotype was first discovered in South Africa by Du Plessis [21]. It is known to occur in the Transvaal region of the country in provinces such as Gauteng, Mpumalanga, Limpopo, and the North West and corresponds to the presence of the *Amblyomma* tick [5]. The pathogenicity of this genotype was tested in cattle, sheep, and goats as well as in mice, and it was discovered that it is one of the most virulent genotype strains ever discovered in South Africa [4]. The Welgevonden genotype is one of the strains trial tested for vaccine development (attenuated vaccine), where it provided 100% protection to small stock in contact with a deadly needle challenge of homologous strain or combined with the Gardel genotype or the Senegal genotype (heterologous strains) [6]. On the other hand, the LemcoT3 genotype originated from Zimbabwe, which is one of the neighboring countries with South Africa [22]. To date, no study has proved that it can be used to improve the current vaccine.

The *E. ruminantium* NWUe2 and NWUe5 *Map1* nucleotide sequences were identical to the GenBank Blaaukrans (AF368000.1), Nyatsanga (U50834.1) and Burkina Faso (AF368001.1) (Table 3). The Blaaukrans genotype was first detected in South Africa in the Eastern Cape Province, where the vector is also found [5]. To date, no study has demonstrated how pathogenic the strain is in domestic ruminants, and it has not been proven whether the current commercial vaccine (infective blood Ball 3 strain) can protect against this genotype strain. The Nyatsanga genotype strain, on the other hand, was first isolated in a cell culture in Zimbabwe with other strains [23,24]. The third strain, the Burkina Faso genotype, originated from West Africa (Burkina Faso) and was isolated from a tissue culture [25]. It has been observed by Martinez et al. [26] that the Burkina Faso isolates are similar to other strains circulating in other African countries. The Burkina Faso genotype has also been shown to improve the inactivated Gardel strain vaccine both in the trial and field challenge when combined with Burkina Faso [27].

The NWUe3 *Map1* nucleotide sequence showed similarities with 5 other nucleotide sequences from the GenBank: er80/1 (EF627980.1), hypothetical transcriptional regulator gene (AY343331.1), Welgevonden Erwe (CR925678.1), Welgevonden Erwo (CR767821.1) and *Cowdria ruminantium* surface protein (U49843.1) (Table 3). The er80/1 is an *E. ruminantium* clone, which was first isolated in West Africa (Gambia) from an *A. variegatum* tick and was never tested for its virulence [28]. The Welgevonden stock of *E. ruminantium,* which was discovered by Du Plessis [21], could grow in vitro in a bovine aorta endothelial cell line, a lamb fetal testis endothelial cell line, and a sheep brain endothelial cell line. The locus containing *Map1* shows that *Map1* is encoded by a multigene family [29]. None of the above-mentioned strains is a real strain of *Ehrlichia* spp., so these similarities achieved could be for other closely related strains or mutations in the area. However, this assumption was not investigated, so future studies are required. The Welgevonden strain was derived from the South African Welgevonden (Erwe), which has been maintained in Guadeloupe (Caribbean Island) since May 1988 in a different cell environment [30]. The Erwe strain was sequenced at CIRAD-Emvt, TA30/G, *Campus International de Baillarguet* in France as a comparative genomic analysis of the recently published parent strain, Erwo, which is the main Welgevonden genotype from South Africa, maintained in Pretoria, Onderstepoort Veterinary Institute (South Africa). It was found that the gene order is highly conserved between the *E. ruminantium* strains [30]. Lastly, the *Cowdria ruminantium* surface protein, which is a clone of Welgevonden strain from South Africa, was sequenced by Brayton et al. [31] in Pretoria, Onderstepoort Veterinary Institute. 

The *E. ruminantium Map1* gene sequences of the Ngaka Modiri Molema District appeared in three different clusters in our phylogenetic analysis. The clusters represented strains that have been previously identified both in South Africa and from other countries. The countries in question have been identified above as west African countries (Burkina Faso and Cameroon) and neighboring countries in southern Africa (Botswana, Zimbabwe and Mozambique). Cross border movement of ticks and animals may be responsible for these findings, but also studies have shown that migratory birds, which could have been potentially infected by *Amblyomma* or other ticks, can introduce *E. ruminantium* in non-endemic areas or countries [32]. 

Genetic diversity of *E. ruminantium* makes it difficult for African countries to control heartwater disease, using vaccines. In South Africa, to date, the available vaccine is the infective sheep blood, containing the Ball3 genotype strain, however, other studies have proved that it does not protect against all the genotypes that are circulating in the field [33]. The diversity noticed in this study may explain why such vaccines have not been very effective but hopefully this data will aid in formulation of better vaccines. 

## 5. Conclusions

The 5 *E. ruminantium* positive amplicons of *Map1* gene shared identities or clustered with more than 13 other different genotypes from the GenBank. Remarkably, the Ball3 genotype strain was not among the 13 GenBank strains which shared similarity with our *E. ruminantium* strains. This observation suggests the possibility that the current commonly used blood vaccine maybe ineffective in the study area. The isolated genotypes from this study can help improve the current vaccine development and are also crucial in understanding the epidemiology and control of heartwater disease. This study will thus serve as a future data reference for more genetic diversity studies and vaccine formulation strategies for the area. 

## Figures and Tables

**Table 1:** Primer sequences for the detection of the *Map1* gene using conventional nested PCR.

Primers	Gene	Sequence (5′-3′)	F/R Primers	Primer Length
NT CT1bi	*Map1* (First phase)	5′-CTCGTAAGAAGTGCGTTAAT-3′ 5′-TTAAAATACAAACCTTCCTCC-3′	external forward external reverse	20 21
LP CT2bis	*Map1* (Second phase)	5′-CTTGGTGTGTCCTTTTCTGA-3′ 5′-CCTTCCTCCAATTTCTATACC-3′	internal forward internal reverse	20 21

**Table 2:** Number of samples collected throughout the 5 municipalities of the Ngaka Modiri Molema District and DNA pool samples screened by PCR *dsbA* and *Map1*.

Municipality	Species	Number of Animals per Species	Number of Ticks	Total Ticks per Municipal	Number of Pools	PCR Positives for *dsbA* Gene (MIR)	Total *dsbA* MIR per Municipal	PCR Positives for *Map1* Gene (MIR)	Total *Map1* MIR per Municipal
Mafikeng	Bovine Ovine Caprine	134 66 50	200 100 75	375	67 33 25	29 (145) 17 (170) 7 (93)	141	2 (10) 1 (10) 1 (13)	11
Ramotshere Moiloa	Bovine Ovine Caprine	100 36 34	150 54 50	254	50 18 17	15 (100) 10 (185) 8 (160)	130	2 (13) 1 (19) 1 (20)	16
Ratlou	Bovine Ovine Caprine	76 14 6	114 20 10	144	38 7 3	8 (70) 4 (200) 2 (200)	97	1 (9) 2 (100) -	21
Ditsobotla	Bovine Ovine Caprine	52 10 6	80 15 8	103	26 5 3	2 (25) 3 (200) 1 (125)	58	- 2 (133) -	19
Tswaing	Bovine Ovine Caprine	- - -	- - -		-	-		-	
Total		584	876		292	106 (121)		13 (15)	

**Table 3:** Results of BLASTn from sequenced nested PCR *Map1* gene product.

Identification	Accession Number	Similar Genotype Strains from GenBank	Query Coverage (%)	Percentage Identity
NWUe1	MZ393498	Welgevonden (AF125274.1)	99.0	99.7
		LemcoT3 (AF125277.1)	99.0	99.5
NWUe2	MZ393499	Blaaukrans (AF368000.1)	98.0	99.0
		Nyatsanga (U50834.1)	99.0	98.6
		Burkina Faso (AF368001.1)	98.0	98.0
NWUe3	MZ393500	Er80/1(EF627980.1)	99.0	99.6
		Hypothetical transcriptional (AY343331.1)	99.0	99.6
		Welgevonden (CR925678.1)	99.0	99.6
		Welgevonden (CR967821.1)	99.0	99.6
		Surface protein (U49843.1)	99.0	99.6
NWUe5	MZ393502	Blaaukrans (AF368000.1)	97.0	100
		Nyatsanga (U50834.1)	99.0	99.6
		Burkina Faso (AF368001.1)	99.0	98.8
NWUe6	MZ393503	Welgevonden (125274.1)	99.0	98.9
		LemcoT3 (AF125277.1)	99.0	98.7

## Data Availability

The data presented in this study are available on request from the corresponding author.

## Supplementary Materials

The following are available online at https://www.mdpi.com/article/10.3390/ani12192511/s1, Figure S1. Sequence polymorphism among nucleotide sequences of MAP-1 from South Africa *Amblyomma haebareum*. Figure S2: Phylogenetic analysis of tick ITS2 gene using the maximum likelihood (ML) method. Table S1. BLASTn of sequence from *dsbA* gene with similar matches with *Ehrlichia ruminantium* from the NCBI.
