# Supplementary material for: Molecular Detection and Genetic Characterization of Ehrlichia ruminantium Harbored by Amblyomma hebraeum Ticks of Domestic Ruminants in North West Province, South Africa
Source: Animals (Basel). 2022 Sep 21;12(19):2511. doi: 10.3390/ani12192511 (PMC9559644; doi:10.3390/ani12192511)
Supplement: Supplementary file 1 [file animals-12-02511-s001.zip › animals-1792945-supplementary.pdf]

Molecular detection and genetic characterization of *Ehrlichia ruminantium* harboured by *Amblyomma hebraeum* ticks of domestic ruminants in North West province, South Africa

Sifiso S. Mnisi[1], Malekoba. B. N. Mphuthi[1], Tsepo Ramatla[2*], lehlohonolo Mofokeng1, Oriel M. M. Thekisoe[2] and Michela Syakalima[1,3]

1. Department of Animal Health, School of Agricultural Sciences, North-West University, Private Bag X2046, Mmabatho, 2735, South Africa. sifisosiza.mnisi@gmail.com; nthabiseng.mphuthi@nwu.ac.za; mich-san65@gmail.com
2. Unit for Environmental Sciences and Management, North-West University, Private Bag X6001, Potchefstroom 2531, South Africa. thekisoe@gmail.com
3. University of Zambia, School of Veterinary Medicine, Disease Control Department, P.O Box 32379, Lusa-ka, Zambia

Table S1. BLASTn of sequence from *dsb*A gene with similar matches with *Ehrlichia ruminantium* from the NCBI.

Identification	similar sequence from GenBank	Query cover (%)	Percentage identity
NWUe1	*Ehrilichia ruminantium*, Kwanyanga. (CP040119.1)	96	99.7
NWUe2	*Ehrilichia ruminantium*, Mara87/7. (CP040118.1)	97	99.4
NWUe3	*Ehrilichia ruminantium*, Nonile. (CP040117.1)	97	99.7
NWUe4	*Ehrilichia ruminantium*, Springbokfontein2. (CP040115.1)	97	99.7
NWUe5	*Ehrilichia ruminantium*, Grootvellei. (CP040120.1)	97	99.4
NWUe6	*Ehrilichia ruminantium*, Grootvellei. (CP040120.1)	97	99.4
NWUe7	*Ehrilichia ruminantium*, Kwanyanga. (CP040119.1)	96	99.4
NWUe8	*Ehrilichia ruminantium*, Grootvellei. (CP040120.1)	94	98.5
NWUe9	*Ehrilichia ruminantium*, Springbokfontein4. (CP040114.1)	90	99.7
NWUe10	*Ehrilichia ruminantium*, Kwanyanga. (CP040119.1)	91	98.8
NWUe11	*Ehrilichia ruminantium*, Springbokfontein4. (CP040114.1)	94	99.1

NWUe12	*Ehrilichia ruminantium*, Kwanyanga. (CP040119.1)	97	99.7
NWUe13	*Ehrilichia ruminantium*, Mara87/7. (CP040118.1)	94	99.4
NWUe14	*Ehrilichia ruminantium*, Grootvellei. (CP040120.1)	96	99.7
NWUe15	*Ehrilichia ruminantium*, Kwanyanga. (CP040119.1)	95	100

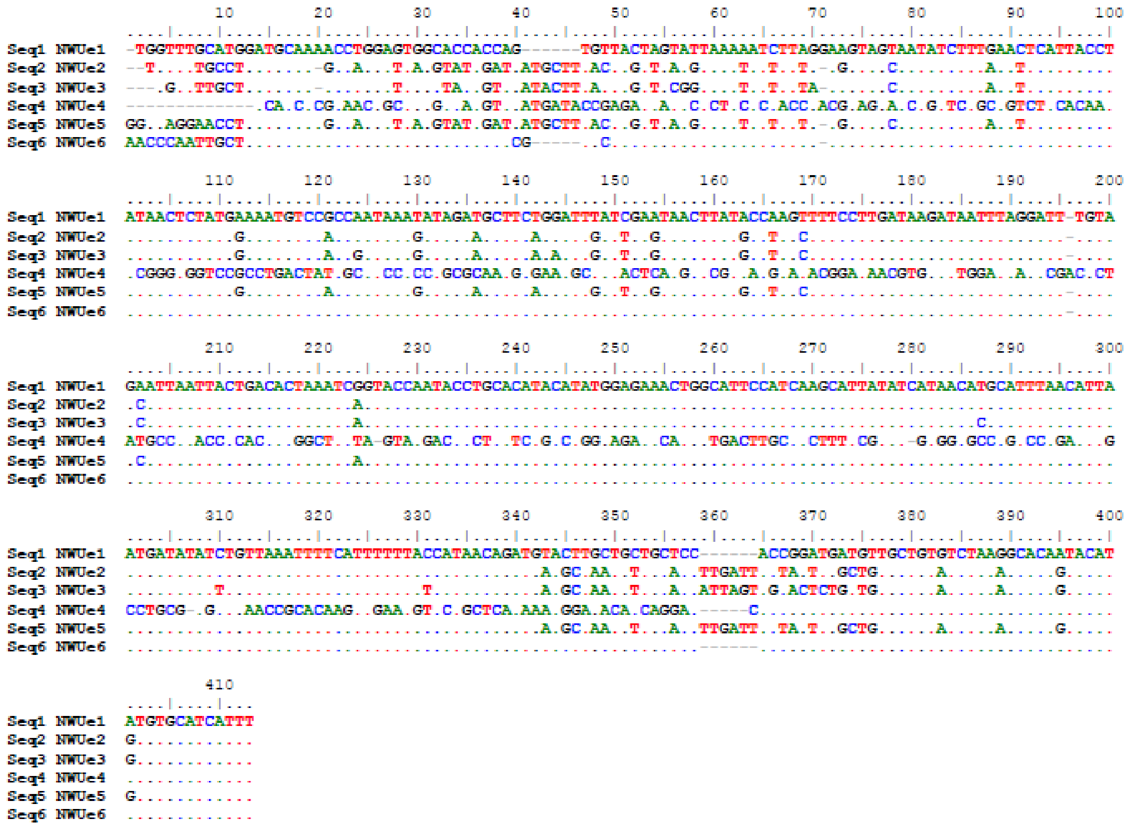

Figure S1: Sequence polymorphism among nucleotide sequences of *map*-1 from South Africa *Amblyomma haebareum*

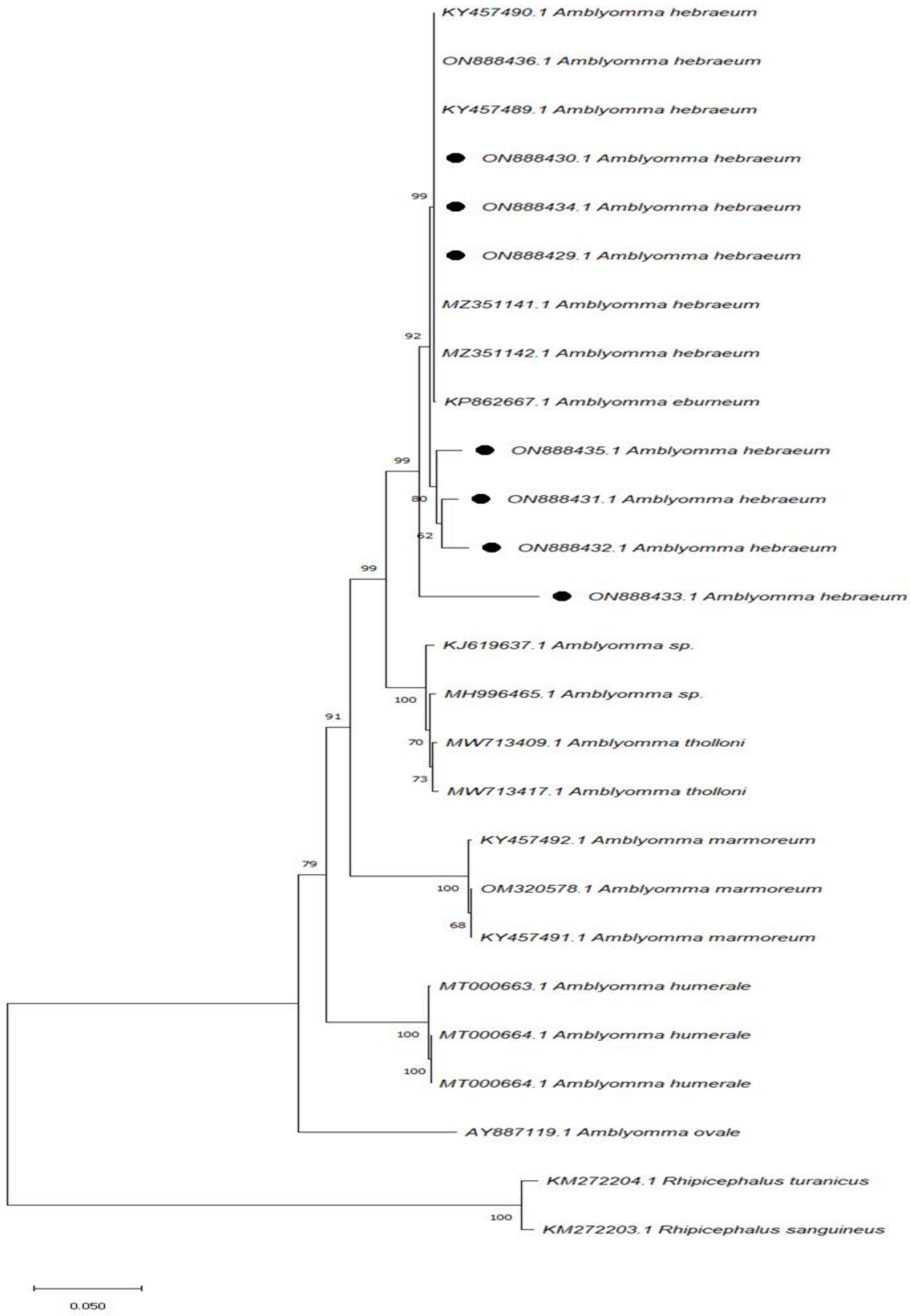

Figure S2: Phylogenetic analysis of tick *ITS2* gene using the Maximum Likelihood (ML) method.
